# Summary, initial outputs and next steps Collaboration for Applied Health Research & Delivery

**DOI:** 10.1186/1753-6561-9-S10-S1

**Published:** 2015-12-18

**Authors:** SB Squire

**Affiliations:** 1 Centre for Applied Health Research & Delivery, Liverpool School of Tropical Medicine, Pembroke Place, Liverpool L3 5QA, UK

## 

The Collaboration for Applied Health Research & Delivery (CAHRD) is an emerging network of organisations and individuals working across the spectrum of applied health research [1] and delivery with the common vision of improving the health of low and middle-income populations through the transformation of health systems. CAHRD has three strategic goals for its first 5 years (2012-2017):

1. The development of a robust human resource base and professional infrastructure

2. The conduct and publication of experience in applied health research and delivery

3. Building strong partnerships and implementing excellent communication strategies.

CAHRD's inaugural Consultation took place in June 2014 attended by almost 170 participants and contributed to all three of these strategic goals. It resulted in a number of strengthened and new collaborative ventures, each aimed at tackling major health challenges for lung health, maternal and newborn health, neglected tropical diseases and health systems over the next 10 to 20 years. The Consultation was the culmination of months of work and preparation centred on establishing creative dialogue and productive joint working across and between a range of international contexts, health topics, and disciplines united by our mission to improve the health of low and middle income populations. This paper summarises the consultation process and conclusions, and identifies initial outputs and next steps.

## Summary of consultation process

The consultation process started by selecting four areas of work where LSTM already has productive global collaborations with coordinating centres in all four of its research departments. This ensured inclusion of those departments expected to have a large portfolio of applied health research and delivery work (the departments of International Public Health and Clinical Sciences) and those more naturally associated with bench science (the departments of Vector Biology and Parasitology). The four workstreams chosen were lung health, maternal and newborn health, neglected tropical diseases, and health systems. Within each workstream a diverse group of individuals tackled the task of writing a discussion paper about the three issues that would be, for them, the major health challenges over the next 10 to 20 years, along with some initial ideas on pathways towards solutions for these challenges. The initial drafts were further developed through sharing and discussions with a wider community of collaborating, global partners from a wide range of institutions and disciplines. The 12 discussion papers were then shared between the four workstreams and thereby subjected to a range of internal and external peer-review. This process started the cross-disciplinary, cross-topic dialogue within LSTM and within existing collaborative networks.

The next stage was to invite a range of external panelists to help inform the initial strategic direction, by using the papers as a starting point for substantive discussion with as many of our colleagues from the Global South as possible. We circulated the papers [2-9] in advance and then set up a range of different discussion formats over a one and a half day meeting in Liverpool. On day 1 there were plenary presentations, introducing the four themes open to all, followed by moderated discussion in small breakout groups. Participants then came together once more in open plenary for the Leverhulme Lecture given by World Bank Director for Health, Nutrition and Population Tim Evans in Liverpool Town Hall. His topic was “Delivery Research” and he argued that the primary knowledge constraints to improving health lie less with “what to do” and much more with “how to do it”, which was in tune with the applied health research and delivery focus of the 12 discussion papers. He further argued that the emergence of a health goal of Universal Health Coverage within the post-2015 Sustainable Development Goals represents a unique opportunity to bring delivery research from the margins to the mainstream of research for health. Day 2 started with a debate in the format of the BBC's *‘Question Time’* programme in which questions submitted by the public are posed to a small panel of selected individuals to discuss with further contributions from the audience. Peter Sissons, one of the UK's most experienced broadcast journalists and a vice-president of LSTM, chaired the debate. This gave the chance for more junior researchers to mix with experienced colleagues on the panel, and allowed for in-depth debate around five questions which we selected from around 60 submitted in advance from across the collaborative networks. The consultation ended with feedback from the group work in an open plenary, with additional discussion on conclusions and next steps.

The consultation process was widely considered a success with lively, energetic discussion that generated new ideas and directions. Mwele Malecela, Director of the National Institute for Medical Research (NIMR) in Tanzania, said “CAHRD is an excellent example of developing policy and research in collaboration between the Global North and the Global South”. Himanshu Bhushan, Deputy Commissioner, Maternal Health, Ministry of Health and Family Welfare, Government of India, echoed Malecela and added that CAHRD might be a way to further efforts in developing countries to “reach the unreached with quality services”. Amuda Baba, representing Institut Pan-Africain de Sante Communautaire (IPASC) and from the fragile Ituri region of Democratic Republic of Congo, said that the consultation had reminded us all to think about “whose health system is it that we are talking about”, and called for local engagement and investment. Jeremiah Chakaya from the Kenya Medical Research Institute (KEMRI), and Ireen Namakhoma from Research for Equity And Community Health (REACH) Trust, Malawi, picked up this call and both look forward to the day when developing countries seriously invest in, and use, applied health research, matching resources generated from developed countries.

## Initial outputs

A CAHRD website has been created which has conference resources including conference documents, presentations and a full recording of Tim Evans' Leverhulme Lecture (http://www.cahrd-network.org/consultation/).

### Relevant blogs and news articles

Several blogs and news articles resulted from the Consultation[10-21]

### Publication of Consultation discussion papers

The overall Introductory paper, setting out the Consultation rationale and process is published in this supplement [3]. Two of the three Lung Health papers are also published in this supplement [3,4]. The third Lung Health paper formed part of the content of a larger paper on indoor air pollution [22]. All three of the Neglected Tropical Diseases papers are published in this supplement [[5-7]. The content of the three Maternal & Newborn Health papers and the subsequent discussion went forward into a number of articles in a supplement on quality of care in the British Journal of Obstetrics and Gynaecology[25,26]. Two of the Health Systems papers are published in this supplement [8,9] and the third was published elsewhere[27]

A number of additional publication outputs were facilitated by the CAHRD Consultation in the fields of Lung Health[23,24] and Maternal & Newborn Health[25,26], and Health Systems[28-32]

### The following areas of enquiry are being taken forward for funding as a result of the CAHRD consultation

• Social competence and influence based approaches for schoolchildren to preventing tobacco smoking

• Integrated Primary care management of Adult Chronic Cough (iPACC) – Health systems modelling for development of interventions.

• Programmatic implementation of shortened drug regimens for multi-drug resistant tuberculosis combined with socio-economic support interventions

• Development and implementation of xenomonitoring tools for human African trypanosomiasis.

• Institutional capacity strengthening in Sierra Leone

• Effect on gender equity of work with close to community providers in a number of different contexts

• Mathematical modelling of the role of new diagnostics in the achievement of the 2020 goals for visceral leishmaniasis

• Mathematical modelling of potential impact of new diagnostics in achieving the 2020 goals for soil-transmitted helminths and schistosomiasis.

## Next steps

Colleagues in CAHRD at the University of Warwick are planning follow up meetings in 2016 to consolidate and develop the research programme and will include direct work in further development of applied health research and delivery proposals for funding. Colleagues in NIMR, Tanzania have volunteered to host the 2018 CAHRD Consultation in collaboration with other individuals and organisations in Africa, including Kenya, Uganda, Malawi and Democratic Republic of Congo.

### Lung Health

• Broaden the scope of the MRC-funded BREATHE Partnership to align synergistically with CAHRD at the applied end of the health research and delivery spectrum.

• Capitalise on opportunities to influence health systems policy and decision making and incorporate influencing policy as a specific strategic aim within BREATHE and CAHRD.

• Integrate approaches for improving lung health with approaches taken by other current and future worksteams to maximise mutually beneficial cross-working towards integrated solutions.

### Maternal and Newborn Health

• Develop discussion paper further to ensure that goals across the continuum of care are captured as sub goals (including Adolescent and Reproductive Health).

• Strengthen our partnerships (internal and external) to continue to conduct high quality operational/health systems research.

• Maintain a strong focus on Quality of Care in the CMNH research strategy and work plans.

### Neglected Tropical Diseases

• Refine and promote use of tools to detect pathogens and vectors (diagnostics, vector sampling methods, data management systems) to enable health systems to detect and respond rapidly to resurgences in NTDs.

• Strengthen quantitative expertise within disease endemic countries to assist in the design, implementation, monitoring and evaluation of locally appropriate control programmes against NTDs.

• Identify how existing national health systems and international programmes implementing preventive chemotherapy might deliver more complex interventions that combine mass-drug administration, case detection and treatment, vector control and disability management.

### Health Systems

• Develop methods and approaches for inter-sectoral action (for example partnership with education, sanitation, transport and gender/social welfare).

• Evaluate enablers at local level for inter-sectoral action including roles for close to community providers.

• Develop and evaluate mechanisms to support equity and access: such health insurance/social protection with a focus on reducing indirect costs and catastrophic health expenditure.

• Explore the opportunities to build platforms for action in fragile and conflict affected states.

We have together set the collaboration off to an enthusiastic start. Our next challenge is to ensure that we now capitalise on the energy and ideas. As we keep our sights firmly on the medium- to long-term future, we need to keep in mind that CAHRD's focus on poorer populations looks set to remain a key priority. As Chris Whitty, Chief Scientific Advisor to the UK's Department for International Development, predicted during the consultation: “While the numbers in the middle classes in developing countries will clearly grow—gross national income (GNI) in developing countries is set to double in the next 10 years—the numbers of skilled health providers is set to expand more slowly. By the laws of supply and demand, these providers will be pulled away from providing quality health care for the poorest”. We really have to collaborate effectively, and energetically, to transform the future of health systems that serve those in greatest need.

## Declarations

This article has been published as part of *BMC Proceedings* Volume 9 Supplement 10, 2015: The 2014 CAHRD (Collaboration for Applied Health Research & Delivery) Consultation. The full contents of the supplement are available online at http://www.biomedcentral.com/bmcproc/supplements/9/S10.
